# Different subtypes of collateral vessels in hemorrhagic moyamoya disease with p.R4810K variant

**DOI:** 10.1186/s12883-020-01884-0

**Published:** 2020-08-19

**Authors:** Peicong Ge, Qian Zhang, Xun Ye, Xingju Liu, Xiaofeng Deng, Jia Wang, Rong Wang, Yan Zhang, Dong Zhang, Jizong Zhao

**Affiliations:** 1 Department of Neurosurgery, Beijing Tiantan Hospital, Capital Medical University, Beijing, 100070 China; 2 China National Clinical Research Center for Neurological Diseases, Beijing, China; 3 Center of Stroke, Beijing Institute for Brain Disorders, Beijing, China; 4 Beijing Key Laboratory of Translational Medicine for Cerebrovascular Disease, Beijing, China; 5 Beijing Translational Engineering Center for 3D Printer in Clinical Neuroscience, Beijing, China; 6 Savaid Medical School, University of Chinese Academy of Sciences, Beijing, China

**Keywords:** Hemorrhgic sites, Collateral vessel, P.R4810 K vaiant, Moyamoya disease, Hemorrhage

## Abstract

**Background:**

The aim of this study was to investigate the hemorrhgic sites and collateral vessels in hemorrhagic MMD with the p.R4810K variant.

**Methods:**

Hemorrhage sites were classified as either anterior or posterior. Collateral vessels were classified into three subtypes according to origin: lenticulostriate anastomosis, thalamic anastomosis, and choroidal anastomosis. Hemorrhage sites and collateral vessels were compared between patients with wild-type p.R4810K variant (GG) and patients with heterozygous p.R4810K variant (GA) after 1:1 propensity score matching.

**Results:**

A total of 130 hemorrhagic MMD patients were included in present study, 21 pairs (42 hemorrhagic hemispheres) were obtained after 1:1 propensity score. In GA group, 16 hemispheres (76.2%) presented anterior hemorrhage, and 5 hemispheres (23.8%) presented with posterior hemorrhage. In GG group, 13 hemispheres (61.9%) presented anterior hemorrhage, and 8 hemispheres (38.1%) presented with posterior hemorrhage. No significant differences were found in hemorrhagic sites between two matched groups (*P* > 0.05). Of 21 hemispheres in GA group, 10 (47.6%) exhibited lenticulostriate anastomosis, 6 (28.6%) thalamic anastomosis, and 6 (28.6%) choroidal anastomosis. Of 21 hemispheres in GG group, 3 (14.3%) exhibited lenticulostriate anastomosis, 5 (23.8%) thalamic anastomosis, and 9 (42.9%) choroidal anastomosis. There was significant difference in lenticulostriate anastomosis between two matched groups (*P* = 0.045). After adjustment the age, sex, and PCA involvement, we found that lenticulostriate anastomosis was associated with p.R4810K variant (OR, 5.995; 95% CI, 1.296–27.737; *P* = 0.022).

**Conclusion:**

Lenticulostriate anastomosis might be associated with p.R4810K variant. Whereas hemorrhagic sites, thalamic anastomosis, and choroidal anastomosis might not be associted withp.R4810K variant.

## Background

Moyamoya disease (MMD) is an uncommon cerebral vascular disease, which is characterized by progressive stenosis of the terminal portions of bilateral internal carotid arteries and/or its main branches associated with compensatory abnormal vascular network at the base of the brain [1, 2]. Intracranial hemorrhage and cerebral ischemia are the two main clinical manifestations of MMD [3, 4].

Intracranial hemorrhage occurs in 21 to 42.4% of MMD [5], which is less common than cerebral ischemia, but intracranial hemorrhage and rebleeding is one of the main causes of death in patients with MMD [6]. The supplementary analysis of Japan Adult Moyamoya (JAM) Trial demonstrated hemorrhagic sites were associated with rebleeding [7], the subtypes of collateral vessels was associated with hemorrhagic sites and rebleeding [8].

The p.R4810K variant in RNF213 was identified as a founder variant with a strong susceptibility in patients with MMD among Japan, Korean, and China [9–11]. Recent studies demonstrated that p.R4810K variants are associated with clinical manifestations, angiographic characteristics, postoperative neovascularization and clinical outcomes [12–14]. The association between p.R4810K variant and hemorrhagic sites, collateral vessels in hemorrhagic MMD remains unknown. The present study attempted to investigate hemorrhagic sites and collateral vessels according to the p.R4810K variant. We conducted 1:1 propensity score matching to reduce the effects of heterogeneity, hemorrhagic sites and collateral vessels were compared between the matched groups.

## Methods

This study was approved by the Ethics Committee of Beijing Tiantan Hospital, Capital Medical University.

### Patients data

From June 2012 to June 2017, all MMD patients admitted into the neurosurgical department at Beijing Tiantan Hospital was screened. MMD was diagnosed according to the Japanese guidelines published in 2012 [15]. Patients who met the following criteria were included in this study: 1) p.R4810K variant was sequenced [16]. 2) Digital subtraction angiography (DSA) was received. 3) experienced intracranial hemorrhage confirmed by CT scan. Informed consent was obtained from all patients or their legal representatives before this study. Demographic information, history of risk factors, hemorrhagic types, and modified Rankin Scale (mRS) scores were collected by trained and certified neurosurgeons.

### Angiographic variables

All angiographic variables were evaluated blindly by two neurosurgeons (more than five-year experience in cerebrovascular disease) blinded to p.R4810K variants. Suzuki stages, posterior cerebral artery (PCA) involvement, hemorrhagic sites, and collateral vessels were evaluated. Discrepancies on the angiographic variables were discussed before a final decision was made.

Hemorrhage sites were classified into two subtypes [7]: anterior hemorrhage and posterior hemorrhage. Hemorrhage in the frontal lobe, anterior temporal lobe, putamen, caudate head, anterior subependymal area, or anterior corpus callosum, is defined as anterior hemorrhage. Hemorrhage in the parietal lobe, occipital lob, thalamus, posterior temporal lobe, posterior subependymal area, or posterior corpus callosum, is defined as posterior hemorrhage.

**Fig. 1:**
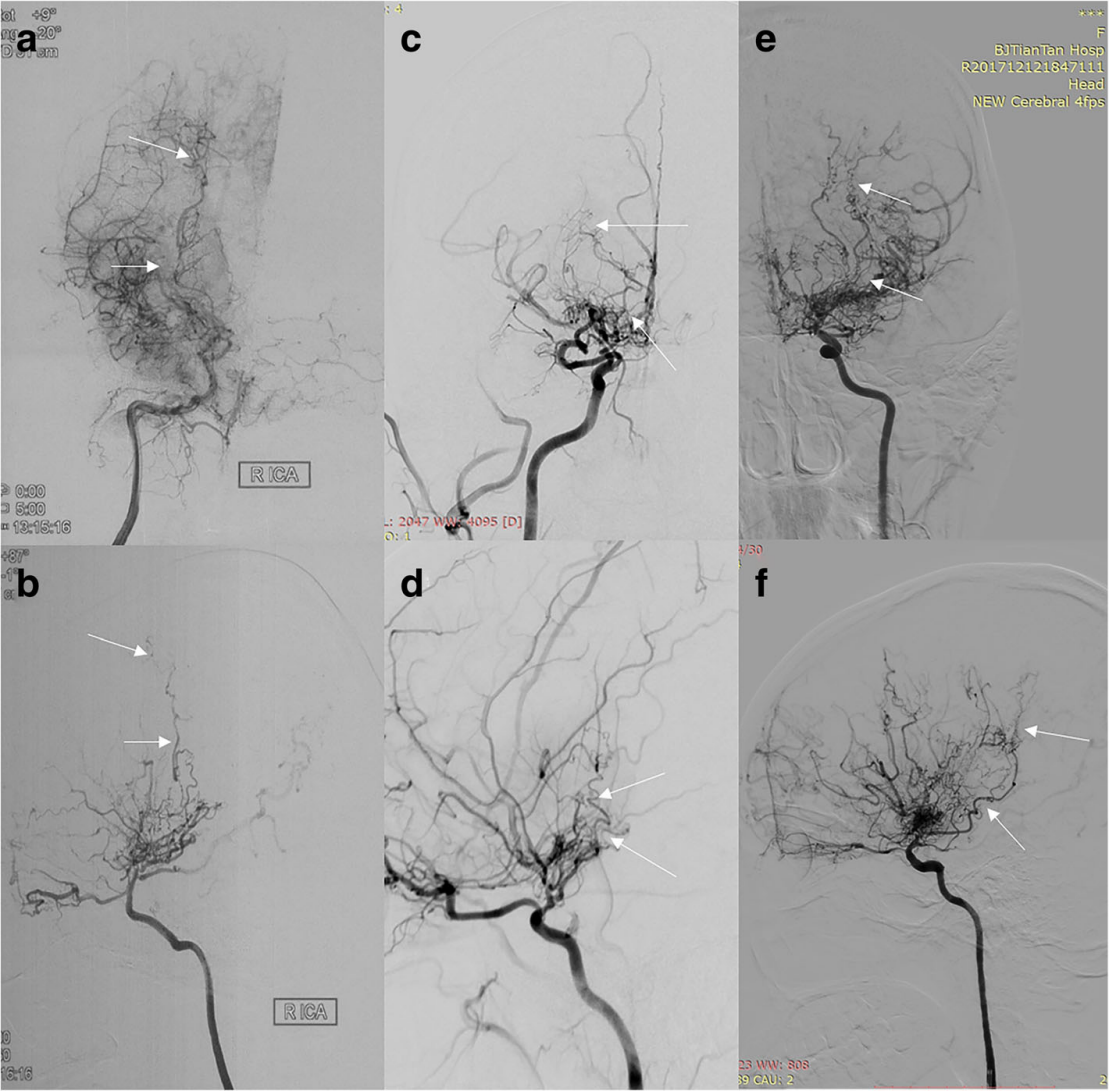
Collateral vessels. **a**, anteroposterior view of lenticulostriate anastomosis; **b**, lateral view of lenticulostriate anastomosis; **c**, anteroposterior view of thalamic anastomosis; **d**, lateral view of thalamic anastomosis; **e**, anteroposterior view of choroidal anastomosis; **f**, lateral view of choroidal anastomosis

Collateral vessels were classified into three subtypes according to origin [8]: lenticulostriate anastomosis, thalamic anastomosis, and choroidal anastomosis. Lenticulostriate anastomosis (Fig. 1a-b): an anastomosis between the lenticulostriate artery and the medial end of the medullary artery with at least 1 artery extending beyond the level of the pericallosal artery in the lateral view. Thalamic anastomosis (Fig. 1c-d**)**: an anastomosis between the thalamic perforator and the medial end of the medullary artery or the insular artery with at least 1 perforator extending beyond the position of the medial posterior choroidal artery in the lateral view. Choroidal anastomosis (Fig. 1e-f): an anastomosis between the choroidal artery and the medial end of the medullary artery with choroidal artery deviated from the level of the lateral ventricle.

### Statistical analysis

Statistical Analyses were conducted by using SPSS 25.0 (IBM; Armonk, New York).

To reduce the imbalance caused by the heterogeneity and retrospective inclusion, including age, sex, family history, hemorrhagic type, history of hypertension, smoking, diabetes, alcohol use, hyperlipidemia, thyroid, and aneurysm, 1:1 propensity score matching (PSM) was carried out. Normal data (age) was compared by using an independent t test. Categorical variables were compared by using Pearson chi-square test, continuity correction test, or Fisher’s exact test as appropriate. The odds ratios (ORs) and 95% confidence intervals (CIs) of p.R4810K variants were calculated according to collateral vessels by using logistic regression model. Two-sided *p* value < 0.05 was considered significant.

## Results

A total of 130 hemorrhagic MMD patients with p.R4810K variant sequenced were included in present study. Of these 130 hemorrhagic MMD patients, 105 (80.8%) patients had wild-type p.R4810K variants (GG), and 25 (19.2%) patients had heterozygous p.R4810K variants.

### Baseline characteristics

Before PSM, patients in GA group were significantly younger than patients in GG group (*p* < 0.001). Patients in GA group exhibited a higher prevalence of family history compared with the GG group (*P* = 0.026). No significant differences were found in the other characteristics, including female/male ratio, hypertension, smoking, diabetes, alcohol use, hyperlipidemia, thyroid disease, aneurysm, hemorrhagic type, and mRS score at admission **(**Table 1**)**. After PSM, 21 pairs (42 hemorrhagic hemispheres) were matched. No significant differences were found in age, female/male ratio, hypertension, smoking, diabetes, alcohol use, hyperlipidemia, thyroid disease, aneurysm, hemorrhagic types, and mRS score at admission.

**Table 1:** Patients characteristics and group comparisons before and after propensity score matching

Characteristics	Before Propensity Score Matching			After Propensity Score Matching		
	GA	GG	p Value	GA	GG	*P* Value
No. of patients	25	105		21	21	
Age, mean ± SD	32.9 ± 11.1	38.3 ± 10.6	**0.026**	33.9 ± 10.1	31 ± 11.0	0.496
Female/male ratio	18/7	62/43	0.232	14/7	13/8	0.747
Family history, n (%)	3 (12.0)	1(1.0)	**0.026**	0 (0.0)	0 (0.0)	1.000
History of risk factors, n (%)						
Hypertension	4 (16.0)	23 (21.9)	0.704	4 (19.0)	3 (14.3)	1.000
Smoking	1 (4.0)	9 (8.6)	0.724	1 (4.8)	1 (4.8)	1.000
Diabetes	1 (4.0)	3 (2.9)	1.000	0 (0.0)	0 (0.0)	1.000
Alcohol use	0 (0.0)	4 (3.8)	1.000	0 (0.0)	0 (0.0)	1.000
Hyperlipidemia	1 (4.0)	3 (2.9)	1.000	1 (4.8)	1 (4.8)	1.000
Thyroid disease	0 (1.5)	2 (2.3)	1.000	0 (0.0)	0 (0.0)	1.000
Aneurysm	3 (12.0)	11 (10.5)	1.000	2 (9.5)	2 (9.5)	1.000
Hemorrhagic type, n (%)						
IVH	15 (60.0)	47 (44.8)	0.323	13 (75.9)	11(66.2)	0.353
ICH with/without IVH	9 (36.0)	47 (44.8)		7 (18.8)	10 (21.8)	
SAH	1 (4.0)	11 (10.5)		1 (5.3)	0 (12.0)	
mRS (< 2) at admission	17 (68.0)	63 (60.0)	0.460	15 (71.4)	13 (61.9)	0.513

*GA* patients with heterozygous genotype, *GG* patients with wild-type genotype, *mRS* modified Rankin Scale

### Angiographic variables

In GA group, 16 hemispheres (76.2%) presented anterior hemorrhage, and 5 hemispheres (23.8%) presented with posterior hemorrhage. In GG group, 13 hemispheres (61.9%) presented anterior hemorrhage, and 8 hemispheres (38.1%) presented with posterior hemorrhage. No significant differences were found in hemorrhagic sites between two matched groups. In GA group, 4 (19.0%) hemispheres were detected no anastomoses, 12 (57.1%) exhibited only 1 type of anastomosis, 5 (23.8%) exhibited 2 types and no hemispheres exhibited all 3 types. In GG group, 5 (23.8%) hemispheres were detected no anastomoses, 15 (71.4%) exhibited only 1 type of anastomosis, 1 (4.8%) exhibited 2 types and no hemispheres exhibited all 3 types. There was no difference in number of anastomosis (*P* > 0.05).

Of 21 hemispheres in GA group, 10 (47.6%) exhibited lenticulostriate anastomosis, 6 (28.6%) thalamic anastomosis, and 6 (28.6%) choroidal anastomosis (Table 2). Of 21 hemispheres in GG group, 3 (14.3%) exhibited lenticulostriate anastomosis, 5 (23.8%) thalamic anastomosis, and 9 (42.9%) choroidal anastomosis. There was significant difference in lenticulostriate anastomosis between two matched groups (*P* = 0.045). After adjustment the age, sex, and PCA involvement, we found that lenticulostriate anastomosis was associated with p.R4810K variant (OR, 5.995; 95% CI, 1.296–27.737; *P* = 0.022**,** Table 3).

**Table 2:** Radiologic profiles of matched couples

	GA (no. [%])	GG (no. [%])	χ2or Z	*P* Value
Hemorrhagic site			1.003	0.317
Anterior	16 (76.2)	13 (61.9)		
Posterior	5 (23.8)	8 (38.1)		
Suzuki stage				
1	0	0		
2	1 (4.8)	1 (4.8)		
3	11 (52.3)	11 (52.3)		
4	7 (33.3)	7 (33.3)		
5	1 (4.8)	1 (4.8)		
6	1 (4.8)	0		
Median score (IQR)	3 (3–4)	3 (3–4)	−0.028	0.978
PCA involvement	5 (23.8)	4 (19.0)	0.000	1.000
Type of anastomosis				
Lenticulostriate	10 (47.6)	3 (14.3)	4.011	**0.045**
Thalamic	6 (28.6)	5 (23.8)	0.123	0.726
Choroidal	6 (28.6)	9 (42.9)	0.933	0.334

*GA* patients with heterozygous genotype, *GG* patients with wild-type genotype, *IQR* interquartile range, *PCA* posterior cerebral artery

**Table 3:** The Associations of p.R4810K Variant with collateral vessels

	GA (no. [%])	GG (no. [%])	OR (95%CI)	*P* Value
Lenticulostriate	10 (47.6)	3 (14.3)	5.995 (1.296–27.737)	**0.022**
Thalamic	6 (28.6)	5 (23.8)	1.209 (0.272–5.375)	0.803
Choroidal	6 (28.6)	9 (42.9)	0.484 (0.124–1.883)	0.295

The multivariate adjustment model was adjusted for age, sex, and PCA involvement

## Discussion

In this retrospective study, we compared hemorrhagic sites and collateral vessels between the matched groups. We found that lenticulostriate anastomosis were associated with p.R4810K variant. Whereas hemorrhagic sites, thalamic anastomosis, and choroidal anastomosis were not correlated with p.R4810K variant.

The p.R4810K variant in RNF213 was identified as a founder variant with a strong susceptibility in MMD patients [17]. The p.R4810K mutation was found in 31.4% MMD patients in China [16], 75.8% MMD patients in Korea [18], and 95.1% MMD patients in Japan [12]. In present study, the incidence of p.R4810K variant in hemorrhagic MMD was 19.2%, which was much lower than the overall incidence. The p.R4810K variant was correlated with phenotype in MMD [12, 16, 18, 19]. Previous study showed that patients with GA or homozygous variant (AA) may have a younger age onset, higher prevalence of family history, more cerebral infarction, and more PCA involvement. The p.R4810K variant may not relate to clinical outcomes in MMD [19, 20]. Although our previous study revealed that patients with GA might associate with better postoperative collateral formation than patients with GG [14], Nomura et al. found that there was no difference in recurrent strokes and functional conditions [20], our previous study also had come to the same conclusion [19]. Recently, we investigated the whether the p.R4810K variant was associated with angiographic characteristics in MMD, the results revealed that patients in GA group may have a different collateral circulation from patients in GG group [13].

In this study, hemorrhagic sites were not correlated with p.R4810K variant. Our previous study showed that anterior hemorrhage may relate to better postoperative collateral formation [21]. And Takahashi et al. found that posterior hemorrhage was associated with a higher rate of recurrent bleeding [7]. Nevertheless, the p.R4810K variant was not associated with clinical outcomes in MMD in previous studies [20]. In present study, no difference was observed in hemorrhagic sites between two matched groups. It might mean that there was no difference in clinical outcomes, which was similar with the previous studies.

Lenticulostriate anastomosis were associated with p.R4810K variant. Whereas thalamic anastomosis, and choroidal anastomosis were not correlated with p.R4810K variant. The dilation and abnormal branching of the anterior choroidal artery was associated with hemorrhagic presentation and the risk of de novo hemorrhage [22–24]. A supplementary analysis of the JAM Trial showed that choroidal anastomosis was associated with posterior hemorrhage and might be a potential source of posterior hemorrhage [8]. In addition, a case control study of the JAM trial showed that different collateral vessels were observed between hemorrhagic and ischemic MMD [25], the thalamic and choroidal anastomosis was more prominent developed in hemorrhagic MMD. The results revealed that direct bypass could reduce the hemodynamic stress to the choroidal and thalamic collaterals [25, 26]. In this study, we found that no difference was observed in thalamic and choroidal anastomosis between two matched groups. It might mean direct bypass may have a role in both patients with GA and GG. Whereas patients with GA had more lenticulostriate anastomosis, recent study showed that lenticulostriate anastomosis in hemorrhagic MMD was less likely to diminish than other collaterals after successful direct bypass [27]. It suggested that patients with GA variants should be given more attention to surgical planning, and the bypass surgery should target the area reached by collateral vessels.

The present study has several limitations. First, although 1:1 PSM was conducted to minimize the effects of heterogeneity in the two groups, potential selection bias might still occur in this retrospective study. Second, the number of matched groups was small, this study was only enrolled patients with GG and GA, hemorrhagic MMD patients with AA variant was not found. High quality study with a larger sample size is needed. Third, only p.R4810K variant in RNF 213 was sequenced, and the effect of the rare variants in RNF213 in hemorrhagic sites and collateral vessels remains unknown.

## Conclusions

Lenticulostriate anastomosis might beassociated with p.R4810K variant. Whereas hemorrhagic sites, thalamic anastomosis, and choroidal anastomosis might not be correlated with p.R4810K variant.

## Data Availability

The datasets supporting the conclusions of this study are available from the corresponding author on reasonable request.

## Abbreviations

JAM: Japan Adult Moyamoya
MMD: Moyamoya disease
PCA: Posterior cerebral artery
PSM: Propensity score matching
